# Systematic Review of Safety of MF59-Adjuvanted Influenza Vaccine in Older Adults

**DOI:** 10.3390/vaccines14040360

**Published:** 2026-04-17

**Authors:** Matias Edgardo Manzotti, Agustin Bengolea, Hebe Vazquez

**Affiliations:** 1 Geriatrics Section, Department of Internal Medicine, German Hospital, Buenos Aires C1118AAT, Argentina; 2 Stamboulian Foundation, Buenos Aires C1012AAI, Argentina

**Keywords:** flu vaccine, safety, adjuvant, older adults, immunosenescence

## Abstract

**Background/Objectives**: Influenza remains a primary cause of severe illness and death in adults over 60. In this group, immunosenescence and existing health conditions make infections more dangerous and traditional vaccines less effective. The MF59-adjuvanted vaccine was specifically designed to overcome these limitations by enhancing the body’s immune activation and antigen presentation. While the vaccine shows clear benefits, some recent concerns regarding vaccine safety have been raised without supporting scientific evidence. Therefore, this systematic review focuses on providing a comprehensive evaluation of its safety outcomes compared to standard vaccines. **Methods**: Following the PRISMA guidelines, a systematic review and meta-analysis were conducted; two researchers independently assessed the eligibility of the studies, and the risk of bias was assessed using RoB2 and ROBINS tools for randomized clinical trials and observational studies, respectively. Pooled risk estimates were calculated using a random-effects model. **Results**: Ten RCTs and three non-RCTs meeting the inclusion criteria were included. No significant differences were found for severe systemic outcomes: Guillain–Barré syndrome (RR 1.01, 95% CI 0.64–1.80) and encephalitis (RR 1.23, 95% CI 0.85–1.78). For other systemic adverse effects, there were no significant differences between adjuvanted and non-adjuvanted vaccines; only myalgia showed a small but significant increase with adjuvanted vaccines (RR 1.35, 95% CI 1.02–1.78) compared with non-adjuvanted vaccines. **Conclusions**: MF59-adjuvanted influenza vaccines have a favorable and well-characterized safety profile in adults aged 60 years and older. Adverse events are predominantly mild and transient, with no evidence of increased risk of serious or immune-mediated outcomes compared with non-adjuvanted vaccines.

## 1. Introduction

Influenza remains a major cause of hospitalization and mortality worldwide and continues to disproportionately affect older adults. Individuals aged 60 years and older are at increased risk of severe influenza-related outcomes [1], including hospitalization, functional decline, exacerbation of chronic diseases, loss of independence, and death. This increased vulnerability is driven not only by chronological aging itself but also by the high prevalence of frailty, multimorbidity, and age-associated physiological decline that characterize this population. Comorbidities and immunosenescence, a progressive remodeling and decline of both innate and adaptive immune functions associated with aging, contribute to reduced vaccine responsiveness, lower antibody production, impaired cellular immunity, and diminished duration of protection after vaccination, increasing susceptibility to infection, raising the risk of complications, and reducing the effectiveness of preventive interventions [2,3,4]. The standard inactivated influenza vaccine is the primary preventive measure, but its effectiveness is limited in this age group [5]. As a consequence of immunosenescence, standard-dose non-adjuvanted influenza vaccines may provide suboptimal protection in older adults, particularly in those with significant comorbidity burden or frailty. To enhance immune responses in elderly adults, the MF59-adjuvanted influenza vaccine has been developed. Oil-in-water emulsions have been used experimentally as adjuvants for over 100 years. MF59 was developed in the 1990s as a squalene-based oil-in-water emulsion and was the first of its kind. Squalene was chosen as the base for this adjuvant because it is a naturally occurring oil synthesized by humans and, therefore, offers low toxicity potential [6]. It has been used in clinical practice since 1997 in its trivalent formulation and, since 2020, in its quadrivalent formulation [7]. MF59 promotes local innate immune activation, enhances recruitment of antigen-presenting cells, and improves the magnitude and breadth of the immune response to influenza antigens. [8]. Clinical studies and post-marketing surveillance have shown a favorable safety profile, with mostly mild and transient local or systemic reactions [9,10,11]. While several studies report benefits of the MF59-adjuvanted vaccine compared with the standard vaccine, others show inconclusive results, likely due to differences in study design, population characteristics, and influenza seasons [12,13,14]. Although the immunogenicity and effectiveness of MF59-adjuvanted influenza vaccines have been evaluated in multiple studies, safety remains a central issue for clinicians, policymakers, and vaccine recipients. In older adults, where the balance between benefits and tolerability is particularly relevant, concerns about adverse events can influence vaccine acceptance, adherence to immunization recommendations, and confidence in public vaccination programs. This is especially important in the current context, where vaccine-related misinformation and disproportionate attention to isolated safety concerns may undermine trust even in long-established preventive interventions. Available evidence suggests that MF59-adjuvanted influenza vaccines are generally well tolerated and associated primarily with mild and transient local or systemic reactions [15]. However, concerns continue to arise regarding the possibility of increased reactogenicity or rare immune-mediated adverse events, such as Guillain–Barré syndrome or neurological complications, despite the absence of consistent scientific evidence supporting a causal association. In addition, the available literature includes both randomized and observational studies with different comparators, surveillance methods, and outcome definitions, which may complicate interpretation when evidence is reviewed in isolation.

This systematic review focuses on providing a comprehensive evaluation of safety outcomes of the MF59-adjuvanted vaccine in adults aged 60 years and older compared to standard vaccines, with particular emphasis on both common adverse events and serious adverse outcomes of clinical relevance, to inform public health strategies and build vaccine confidence.

## 2. Materials and Methods

- **Protocol and Registration**

The study protocol was registered in PROSPERO (ID: CRD420261288915).

The review process followed the Preferred Reporting Items for Systematic Reviews and Meta-Analyses (PRISMA) guideline [16].

- **Eligibility Criteria**
  The review focused on the following PICO question:
  ○ Patients (P): Adults aged ≥60 years, including hospitalized, community-dwelling, and institutionalized (e.g., long-term care and nursing home) individuals, with or without comorbidities
  ○ Intervention (I): Trivalent (TIV) or quadrivalent (QIV) MF59-adjuvanted influenza vaccine
  ○ Comparator (C):
    − Primary comparator: Standard-dose influenza vaccine
    − Secondary comparator: High dose influenza vaccine (HD) or other formulations
  ○ Outcomes (O):
    − Safety: Adverse events of any type and serious adverse events, as defined in primary studies. This systematic review does not include continuous outcome analysis.

- **Inclusion criteria**
  ○ Study design: RCTs and observational studies; the rationale for, including both RCTs and observational studies was due to their complementary roles in safety assessment (controlled conditions versus real-world detection of rare events).
  ○ Population: Adults ≥ 60 years, including high-risk groups (hospitalized, community-dwelling, and institutionalized)
  ○ Intervention: MF59-adjuvanted influenza vaccines (TIV or QIV)
  ○ Comparator: Non-adjuvanted influenza vaccines or no vaccination
  ○ Outcomes: Safety outcomes as defined above
  ○ Language: No restriction
  ○ Publication period: From vaccine availability to November 2024

- **Exclusion criteria**
  ○ Studies not reporting outcomes of interest
  ○ Non-human studies
  ○ Editorials, letters, commentaries, case reports, and narrative reviews

- **Literature Search**
  ○ Electronic search
    A search was conducted in the Cochrane Database of Systematic Reviews (CDSR), Database of Abstracts of Reviews of Effectiveness (DARE), PubMed, LILACS, CINAHL, PsycINFO, EMBASE, and the online database ClinicalTrials.gov. The identification of primary studies was complemented with a specific search in the PubMed database. All searches covered the period from the vaccine availability dates until November 2024, with no restrictions on publication date and status. Search strategy: (“adjuvants vaccine”[Supplementary Concept] OR “adjuvants vaccine”[All Fields] OR “vaccine adjuvant”[All Fields] OR “adjuvants, vaccine”[MeSH Terms] OR (“adjuvants”[All Fields] AND “vaccine”[All Fields]) OR “vaccine adjuvants”[All Fields] OR (“vaccine”[All Fields] AND “adjuvant”[All Fields])) AND (“influenza, human”[MeSH Terms] OR (“influenza”[All Fields] AND “human”[All Fields]) OR “human influenza”[All Fields] OR “flu”[All Fields]) AND (“adult”[MeSH Terms] OR “adult”[All Fields] OR “adults”[All Fields] OR “adult s”[All Fields]).
  ○ Other search sources
    To ensure the identification of articles that may not have been detected by the search strategy or are not available in the included databases, the following sources of information were included:
    ○ RCTs included in other relevant systematic reviews, identified through a manual review.
    ○ Manual review of references from included studies.

- **Study Selection**

**Figure 1:**
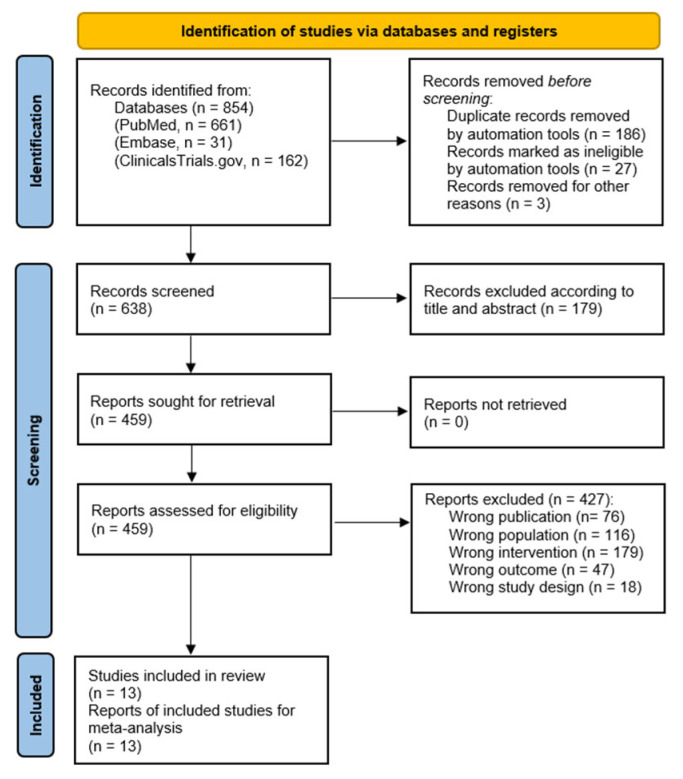
PRISMA flow diagram.

Two independent reviewers (HV and MM) screened titles and abstracts according to the predefined eligibility criteria. Full texts of potentially eligible studies were reviewed independently, with discrepancies resolved by discussion or consultation with a third reviewer (AB). Exclusion reasons and the overall selection process are documented in a PRISMA flow diagram (Figure 1).

- **Data Extraction**
  Data extraction was performed independently by two reviewers (HV and MM) using standardized forms. Extracted information included:
  ○ Study characteristics (author, year, country, and design)
  ○ Participant demographics (age, sex, and comorbidities)
  ○ Intervention and comparator details
  ○ Reported outcomes, with a focus on safety for this manuscript: adverse events of any type and serious adverse events, according to definitions in primary studies.
  Discrepancies were resolved by discussion or consulting a third reviewer (AB). Data were entered into pre-designed spreadsheets for analysis.

- **Publication Bias**

Publication bias was evaluated using funnel plots and Egger’s test. Egger’s test results indicated no statistically significant evidence of publication bias, with all *p*-values exceeding 0.05.

- **Risk of Bias/Quality Assessment**
  Two reviewers independently assessed the risk of bias of the included studies using validated tools.
  ○ RCTs: Cochrane Risk of Bias 2 (RoB 2) tool [17]. The five domains of bias considered in this tool were bias due to the randomization process, bias due to deviations from intended interventions, bias due to missing outcome data, bias in outcome measurement, and bias in selection of the reported result.
  ○ Observational studies: We evaluated methodological quality and risk of bias using the Risk of Bias in Non-Randomized Studies of Interventions (ROBINS-I) tool [18]. This framework assesses bias in seven domains. The domains assessed by this tool were: confounding factors, classification of interventions, participant selection, study selection, outcome measurement, deviations from planned interventions, missing data, and selection of reported outcomes. Two reviewers independently conducted the assessments. Any disagreement was resolved by consensus, moderated by a third reviewer. Each domain was rated as having low, moderate, serious, or critical risk of bias according to the ROBINS-I criteria. Although risk of bias was assessed at the study level, the certainty of evidence for each outcome was not formally evaluated using the GRADE approach.
  Results are summarized in tables within the manuscript.

- **Data Synthesis**

We conducted a meta-analysis through the Web-Based Tool metaanalysisonline platform [19], integrating multiple statistical approaches, including those recommended by the Cochrane Collaboration [20]. This involved selecting studies with significant homogeneity in design, population, interventions, comparators, and reported outcome measures. When studies were considered clinically homogeneous, results were pooled using a random-effects model with the Mantel–Haenszel method to estimate risk ratios (RRs) with 95% confidence intervals. Absolute risk differences (ARDs) and number needed to harm (NNH) were calculated based on pooled event rates to enhance clinical interpretability of statistically significant outcomes. Safety outcomes were summarized narratively and, where possible, quantitatively. Adverse events were classified according to definitions reported in primary studies. Subgroup analyses were planned if sufficient data were available.

- **Effect measures**

In the analysis of dichotomous outcomes, we expressed the estimation of the therapeutic impact of the intervention using risk measures along with the 95% confidence interval (CI). Analysis was performed using a random-effects model with the Mantel-Haenszel method to compare the risk ratio.

- **Heterogeneity assessment**

Variations in the treatment effect among the different included clinical trials were assessed using the X2 test (Q statistic) and the I2 statistic. Statistically significant heterogeneity was considered when the *p*-value was <0.1. Potential sources of heterogeneity include differences in study populations, vaccine formulations, follow-up duration, and adverse event definitions.

- **Subgroup analysis**

We explored the following potential effect modifiers: (1) Risk of bias; we anticipated bigger beneficial effects in high-risk of bias studies. (2) Type of study; we anticipated bigger beneficial effects in non-RCT studies. (3) Population risk; we anticipated bigger beneficial effects in high-risk individuals. To assess the possibility of a subgroup effect, we employed the Instrument for Credibility Assessment of Effect Modification (ICEMAN) [21,22] designed to evaluate the credibility of a claim of effect modification, also known as a subgroup effect, statistical interaction, moderation, or heterogeneity of treatment effects. Subgroup analyses were planned; however, due to the limited number of studies contributing to each outcome, subgroup results were interpreted cautiously. Formal interaction tests were not emphasized to avoid overinterpretation of underpowered comparisons.

Sensitivity analyses using a leave-one-out approach were conducted for outcomes with moderate heterogeneity to assess the robustness of pooled estimates.

## 3. Results

### 3.1. Search Results

Through the search strategy, 638 references were identified for screening by title and abstract. Of these, 459 references were included for full-text evaluation. Finally, 13 trials incorporated safety data [23,24,25,26,27,28,29,30,31,32,33,34,35], 10 RCTs, and 3 non-RCTs. Exclusion reasons for clinical trials and the selection process were recorded in the PRISMA flow diagram (Figure 1). Results were interpreted with consideration of study design and heterogeneity, rather than assuming equivalence across designs.

### 3.2. Description of Included Studies

The included studies addressed diverse populations and different vaccine comparators. In the 13 studies with systematic safety assessment, 3 observational studies and 10 RCTs, solicited adverse events were recorded during the first 7–14 days after vaccination; unsolicited adverse events were generally monitored for up to 28 days; and serious adverse events were followed from vaccination for approximately 6 to 12 months, depending on the protocol. The characteristics of the included studies for analysis are shown in Table 1.

### 3.3. Results of Publication Bias Assessment

The funnel plot does not indicate a potential publication bias. The Egger’s test does not support the presence of funnel plot asymmetry (intercept: 2.17, 95% CI: −0.3–4.65, t: 1.721, *p*-value: 0.113), indicating no strong evidence of publication bias overall. The limitations of these methods are particularly relevant given the small number of studies available for certain outcomes (Supplementary File S1).

### 3.4. Risk of Bias Assessment

Although certain considerations were identified in some domains of the Risk of Bias in Non-Randomized Studies of Interventions (RoBINS-I V2) tool, the overall interpretation of the risk of bias in the primary studies was low to moderate for adverse events in non-RCTs [29,30,33]; the risk of bias in RCTs was heterogeneous. Of the 10 RCTs, 4 had a low risk of bias [24,26,31,32], 4 a moderate risk [25,27,28,35], and 2 a high-risk of bias [23,34]. Detailed risk of bias assessment is shown in Table 1.

### 3.5. Safety Results

As safety effects, the primary studies include diverse effects, from local to systemic effects.

#### 3.5.1. Systemic Effects

The main systemic effects that we were most interested in analyzing in this review were Guillain-Barré syndrome, encephalitis or encephalomyelitis, seizures, nephritis, and vasculitis. The evidence for rare adverse events is limited and should be interpreted with caution.

We found no studies that met the inclusion criteria and presented data on seizures, nephritis, and vasculitis.

**Figure 2:**
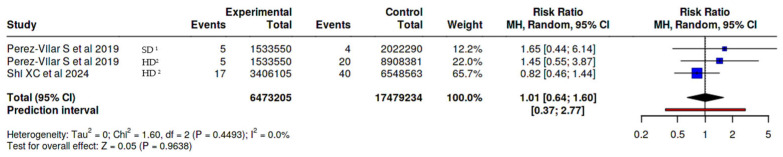
Risk of Guillain-Barré Syndrome, MF59-adjuvanted influenza vaccine vs. all other non-adjuvanted influenza vaccines [29,33]. ^1^ SD standard dose, ^2^ HD high dose.

1. Guillain-Barré syndrome (GBS)
  When we analyzed the data on GBS, we found no significant differences in the risk of developing this syndrome related to the use of adjuvanted vaccines. Of the 2 observational studies that reported on this outcome, one compared the unadjuvanted vaccine, and the other compared both the unadjuvanted and high dose vaccines.
  - MF59-adjuvanted influenza vaccine vs. all other non-adjuvanted influenza vaccines.
  - Altogether 3 observational studies were analyzed with a total of 6,473,205 subjects in the MF59-adjuvanted influenza vaccine experimental cohort and 17,479,234 subjects in the control cohort (non-adjuvanted influenza vaccines). The overall risk ratio was 1.01 with a 95% confidence interval of 0.64–1.6 (Figure 2).

2. Encephalitis

**Figure 3:**
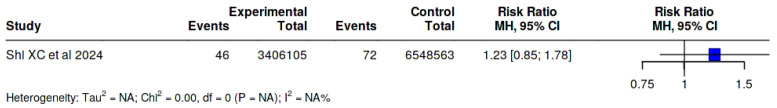
Risk of encephalitis, MF59-adjuvanted influenza vaccine vs. HD vaccine [33].

Only 1 study was found (comparing an adjuvanted vaccine versus a high dose vaccine) and was analyzed with a total of 3,406,105 subjects in the MF59-adjuvanted influenza vaccine experimental cohort and 6,548,563 subjects in the control cohort (HD vaccine). The overall risk ratio is 1.23 with a 95% confidence interval of 0.85–1.78 (Figure 3). No increased risk was observed; however, rare adverse events cannot be definitively excluded.

**Figure 4:**
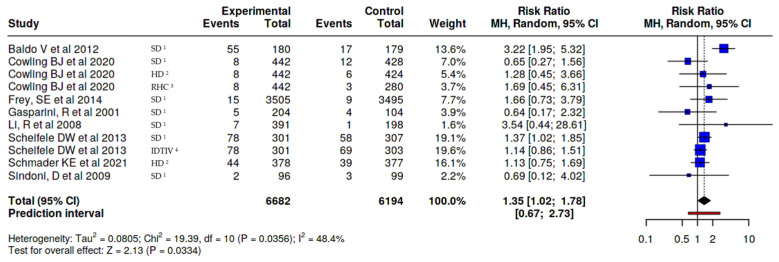
Risk of muscle pain/myalgia, MF59-adjuvanted influenza vaccine vs. all other non-adjuvanted vaccines [23,24,26,27,28,31,32,34]. ^1^ SD standard dose, ^2^ HD high dose, ^3^ RHC recombinant hemagglutinin vaccine, and ^4^ IDTIV intradermal trivalent influenza vaccine.

**Figure 5:**
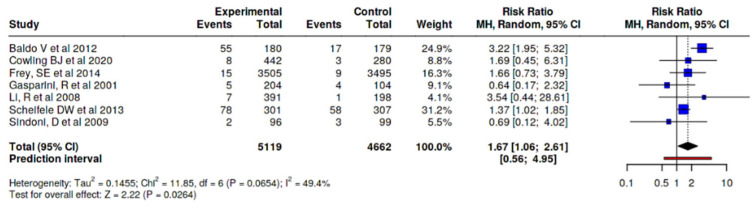
Risk of muscle pain/myalgia, MF59-adjuvanted influenza vaccine vs. non-adjuvanted trivalent or tetravalent SD vaccines [23,24,26,27,28,31,34].

**Figure 6:**
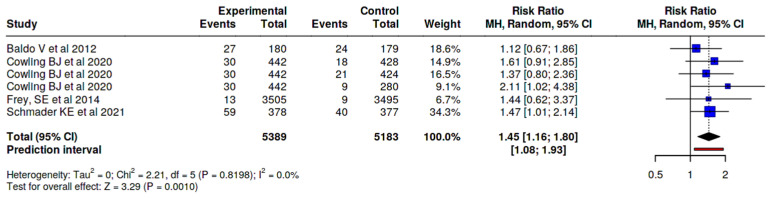
Risk of fatigue, MF59-adjuvanted influenza vaccine vs. all other non-adjuvanted vaccines [23,24,26,32].

**Figure 7:**
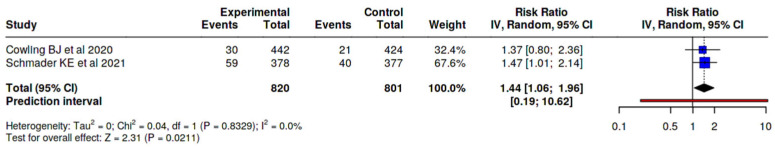
Risk of fatigue, MF59-adjuvanted influenza vaccine vs. HD vaccine [24,32].

3. Other systemic effects
  - Myalgia
    In myalgia/muscle pain outcome, a difference was observed between those who received the adjuvanted vaccine, with a small increased risk (RR 1.35, 95% CI 1.02–1.78), versus any other type of vaccine (Figure 4), and RR 1.67 (95% CI 1.06–2.61) versus non-adjuvanted trivalent or tetravalent SD vaccines, corresponding to an absolute risk difference of approximately 3.7%, resulting in an estimated number needed to harm (NNH) of 27, depending on baseline risk assumptions. This indicates that one additional adverse event would be expected for every 27 individuals vaccinated with the MF59-adjuvanted vaccine compared to the SD vaccine. (Figure 5).
  - Fatigue
    In fatigue outcome, a difference was observed between those who received the adjuvanted vaccine, with an increased risk (RR 1.45, 95% CI 1.16–1.80), versus any other type of vaccine (Figure 6). No significant differences were observed when the adjuvanted vaccine was compared with SD non-adjuvanted vaccines (RR 1.34, 95% CI 0.95–1.89). However, when compared with the high dose vaccine, a higher risk of fatigue was observed (RR 1.44, 95% CI 1.06–1.96). For fatigue, the increased relative risk corresponded to an absolute risk difference of approximately 4–5%, resulting in an estimated number needed to harm (NNH) of approximately 20–25, depending on baseline risk assumptions. (Figure 7).

We found other systemic effects reported more frequently, such as fever, headache, arthralgia, chills, diarrhea, itching, malaise, tiredness, vomiting, and sleep disturbance. No significant difference was found in the outcomes of any of these adverse effects between patients who received the adjuvanted vaccine versus any other vaccine (Supplementary File S2).

#### 3.5.2. Local Effects

**Figure 8:**
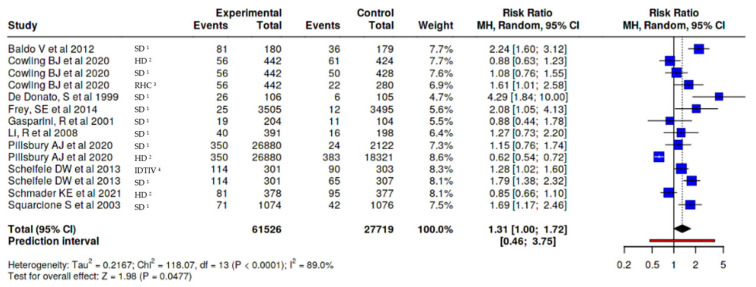
Risk of injection site pain, MF59-adjuvanted influenza vaccine vs. all non-adjuvanted vaccines [23,24,25,26,27,28,30,31,32,35]. ^1^ SD standard dose, ^2^ HD high dose, ^3^ RHC recombinant hemagglutinin vaccine, and ^4^ IDTIV intradermal trivalent influenza vaccine.

**Figure 9:**
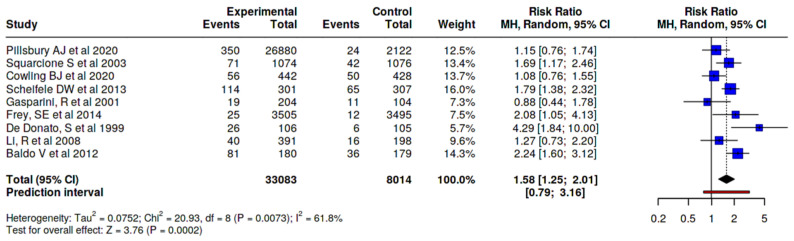
Risk of injection site pain, MF59-adjuvanted influenza vaccine vs. non-adjuvanted SD vaccines [23,24,25,26,27,28,30,31,35].

**Figure 10:**
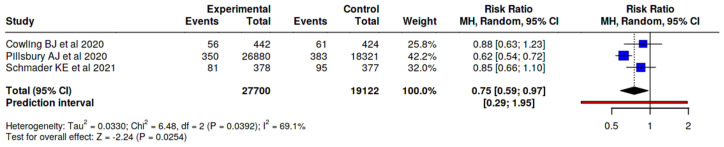
Risk of injection site pain, MF59-adjuvanted influenza vaccine vs. HD vaccine [24,30,32].

Regarding local adverse effects, we found that the adjuvanted vaccine, compared to the altogether non-adjuvanted vaccine, presents an injection site pain RR 1.31, 95% CI 1.00–1.72 (Figure 8); compared to the non-adjuvanted SD vaccines, it presents a higher risk of injection site pain (RR 1.58, 95% CI 1.25–2.01) (Figure 9). When analyzing this effect versus the high dose vaccine, it was observed that the risk of injection site pain is lower with the adjuvanted vaccine (RR 0.75, 95% CI 0.59–0.97) (Figure 10).

### 3.6. Subgroup Analysis

No credible subgroup effects were identified. Exploratory subgroup analyses did not identify credible effect modification; however, these findings are limited by the small number of studies per subgroup. Leave-one-out sensitivity analyses did not materially change the direction or magnitude of the pooled estimates.

## 4. Discussion

This systematic review provides a comprehensive and up-to-date evaluation of the safety profile of the MF59-adjuvanted influenza vaccine in adults aged 60 years and older. Despite many years of experience (since 1997), there is no modern comprehensive synthesis of safety data in the elderly population. This work differs from previous studies by focusing on rare serious adverse events and providing a detailed comparison with different types of vaccines. Previous meta-analyses [12] have primarily focused on efficacy and immunogenicity rather than safety in the elderly. By integrating evidence from randomized controlled trials and large observational studies with systematic adverse event surveillance, our findings consistently demonstrate that MF59-adjuvanted influenza vaccines are well tolerated in older adults, with predominantly mild and transient adverse events and no evidence of an increased risk of serious or immune-mediated adverse outcomes. The relevance of these findings is underscored by the well-recognized impact of immunosenescence on influenza susceptibility and vaccine responsiveness in older populations. Age-related immune dysfunction reduces the effectiveness of standard-dose inactivated influenza vaccines, thereby increasing the need for enhanced vaccine formulations such as adjuvanted or high dose vaccines. In this context, safety considerations are central to vaccine acceptance, regulatory decision-making, and public confidence. Our results support the use of MF59-adjuvanted vaccines as a safe preventive strategy for this high-risk population. Across the included studies, systemic adverse events such as fever, headache, arthralgia, chills, gastrointestinal symptoms, and fatigue were generally comparable between MF59-adjuvanted vaccines and non-adjuvanted formulations. When differences were observed, they were modest and clinically mild. A small but statistically significant increase in myalgia and fatigue was identified in some comparisons, particularly versus non-adjuvanted vaccines or high dose formulations, respectively. Importantly, these symptoms were self-limited and did not translate into increased rates of serious adverse events or vaccine discontinuation. Local reactogenicity, particularly injection-site pain, was more frequently reported with MF59-adjuvanted vaccines compared with standard non-adjuvanted formulations but occurred less often than with high dose influenza vaccines. This gradient of local reactogenicity across enhanced vaccine platforms has been consistently reported in previous trials and post-marketing studies and reflects expected differences in immune stimulation rather than safety concerns. From a clinical perspective, the increased local reactogenicity associated with MF59-adjuvanted vaccines appears acceptable when balanced against the benefits of improved immunogenicity and potential effectiveness in older adults.

A key strength of this review is the focused evaluation of adverse events of special clinical interest in older populations, including Guillain–Barré syndrome, encephalitis or encephalomyelitis, vasculitis, nephritis, and seizures. One of the most clinically reassuring findings of this review is the absence of evidence suggesting an increased risk of serious neurological or immune-mediated adverse events associated with MF59-adjuvanted influenza vaccines. Specifically, no significant increase was observed for Guillain–Barré syndrome or encephalitis in the available comparative analyses, and no eligible studies were identified showing excess risk for seizures, nephritis, or vasculitis. Although some of these outcomes are extremely rare and difficult to study even in large surveillance systems, and rare adverse events cannot be definitively excluded due to limitations in sample size and event frequency, the consistency of the available evidence across observational and randomized data is reassuring. This is especially important because rare neurological events often receive disproportionate attention in public discourse and may contribute to vaccine hesitancy even when causality is unsupported.

At the level of more common adverse outcomes, the overall pattern observed was biologically plausible and clinically acceptable. MF59-adjuvanted vaccines were associated with slightly higher rates of some expected local and systemic reactions compared with standard non-adjuvanted formulations, particularly injection-site pain and, in some analyses, myalgia or fatigue. However, these differences were generally modest and did not translate into an increased burden of serious adverse events. These estimates should be interpreted cautiously, as they are based on pooled event rates and may vary depending on baseline risk across populations. Absolute risk estimates suggest that, although some reactions were more frequent, the magnitude of these differences was small. The estimated NNH values indicate that a relatively large number of individuals would need to be vaccinated for one additional adverse event to occur, supporting the limited clinical impact of these findings.

Furthermore, when compared with high dose influenza vaccines, the MF59-adjuvanted vaccine showed a comparable or in some cases more favorable profile for selected outcomes, such as local pain and fever.

This pattern is consistent with the known mechanism of action of MF59. As a squalene-based oil-in-water adjuvant, MF59 is designed to enhance the innate immune response at the injection site, promote local chemokine production, and facilitate recruitment and activation of antigen-presenting cells. As a result, a modest increase in short-term local or systemic reactions is expected and may reflect effective immune stimulation rather than pathological toxicity. From a clinical standpoint, these reactions are generally self-limited and manageable and should be interpreted in the broader context of the potential benefits of improved immunogenicity and protection in a high-risk population. This review has several strengths, including a broad and systematic search strategy without language restrictions, inclusion of diverse study designs and populations, assessment across multiple influenza seasons, and the use of validated risk-of-bias tools for both randomized and non-randomized studies. Additionally, the application of quantitative synthesis where appropriate, complemented by structured narrative analysis, allowed for a balanced interpretation of heterogeneous safety outcomes. To account for the observed heterogeneity, we examine several contributing factors, such as variations in population profiles, diversity in outcome definitions, and differences in follow-up periods. Nevertheless, certain limitations should be acknowledged. Although serious adverse events were followed for up to 6–12 months in most studies, the included trials and observational analyses may not have been powered to detect extremely rare events. The lack of standardized definitions for adverse events, reporting practices, and follow-up durations across studies may have introduced heterogeneity in some outcomes. Furthermore, standardized adjudication of suspected immune-mediated adverse events was not uniformly applied across studies. This should highlight the need for more standardized safety notifications in future investigations. Subgroup analyses were limited by the small number of studies and should be interpreted with caution, as they may be underpowered to detect true effect modification. Sensitivity analyses suggested that the main findings were robust to the exclusion of individual studies.

Despite these limitations, the findings of this review have important implications for public health and immunization policy. Influenza continues to impose a substantial burden of morbidity, mortality, and functional decline among older adults, particularly those with comorbidities, frailty, or residence in long-term care facilities. Enhanced influenza vaccines represent a critical strategy to mitigate this burden.

Our findings are also relevant for clinicians and vaccination program decision-makers when discussing enhanced influenza vaccine options for older adults. In many settings, healthcare professionals and patients must choose among multiple age-targeted influenza vaccine platforms, including MF59-adjuvanted, high dose, recombinant, or cell-based formulations. While effectiveness and immunogenicity remain key considerations, safety and tolerability often play an important role in patient acceptance and in implementation at the programmatic level.

Overall, the evidence synthesized in this review indicates that MF59-adjuvanted influenza vaccines have a favorable and clinically acceptable safety profile in adults aged 60 years and older, supporting their role as an important preventive strategy in the care of older populations.

## 5. Conclusions

This systematic review demonstrates that MF59-adjuvanted influenza vaccines have a favorable and well-characterized safety profile in adults aged 60 years and older. Adverse events are predominantly mild and transient, with no evidence of increased risk of serious or immune-mediated outcomes compared with standard-dose or high dose influenza vaccines. Continued post-marketing surveillance and comparative effectiveness research across enhanced vaccine platforms remain essential to optimize influenza prevention strategies and sustain public and professional confidence in vaccination programs for aging populations.

## Figures and Tables

**Table 1:** Characteristics of the included studies.

Autor and Publication Year	Study Design	Country	Intervention	Comparator	Risk of Bias	Funding Source
Baldo, V., 2012 [23]	RCT	Italy	aTIV ^1^	TIV ^2^	High	University and Novartis
Cowling, B.J., 2020 [24]	RCT	Hong Kong	aTIV ^1^	QIV ^3^, TIVHD ^4^, RHC ^5^	Low	Government
De Donato, S., 2019 [25]	RCT	Italy	aTIV ^1^	TIV ^2^	Moderate	Government and Novartis
Frey, S.E., 2014 [26]	RCT	Colombia, Panama, Filipinas, USA	aTIV ^1^	TIV ^2^	Low	Novartis
Gasparini, R., 2001 [27]	RCT	Italy	aTIV ^1^	TIV ^2^	Moderate	No declared
Li, R., 2008 [28]	RCT	China	aTIV ^1^	TIV ^2^	Moderate	Novartis
Perez-Vilar, S., 2019 [29]	Observational study, multilayered approach to active safety surveillance	USA	aTIV ^1^	HD ^2^, QIV ^3^	Low	Government
Pillsbury, A.J., 2020 [30]	Cohort study of self-reported survey data	Australia	aTIV ^1^	TIVHD ^4^, QIV ^3^	Low	Government
Scheifele, D.W., 2013 [31]	RCT	Canada	aTIV ^1^	TIV ^2^, IDTIV ^6^	Low	Novartis, Sanofi, and government
Schmader, K.E., 2021 [32]	RCT	USA	aTIV ^1^	TIVHD ^4^	Low	Government and University
Shi, X.C., 2024 [34]	Self-controlled case series design	USA	aTIV ^1^	any available vaccine HD ^7^, SD ^8^	Moderate	Government
Sindoni, D., 2009 [35]	RCT	Italy	aTIV ^1^	TIV ^2^	High	No declared
Squarcione, S., 2003 [12]	RCT	Italy	aTIV ^1^	TIV ^2^	Moderate	Aventis, Pasteur, and MSD

^1^ aTIV adjuvant trivalent influenza vaccine, ^2^ TIV trivalent influenza vaccine, ^3^ QIV quadrivalent influenza vaccine, ^4^ TIVHD trivalent influenza vaccine high dose, ^5^ RHC recombinant hemagglutinin vaccine, ^6^ IDTIV intradermal trivalent influenza vaccine, ^7^ HD high dose, ^8^ SD standard dose.

## Data Availability

The original contributions presented in this study are included in the article/Supplementary Materials. Further inquiries can be directed to the corresponding author.

## Supplementary Materials

The following supporting information can be downloaded at: https://www.mdpi.com/article/10.3390/vaccines14040360/s1, Table S1: PRISMA 2020 checklist. Figure S1: publication bias assessment. Figure S2: Risk of Fever. Figure S3: Risk of Fever vs. HD. Figure S4: Risk of Headache. Figure S5: Risk of Arthralgia. Figure S6: Risk of Chills. Figure S7: Risk of Diarrhea. Figure S8: Risk of Itching. Figure S9: Risk of Malaise. Figure S10: Risk of Tiredness. Figure S11: Risk of Vomiting. Figure S12: Risk of Sleep Disturbance.

## Abbreviations

The following abbreviations are used in this manuscript:

aTIV	adjuvant trivalent influenza vaccine
TIV	trivalent influenza vaccine
QIV	quadrivalent influenza vaccine
TIVHD	trivalent influenza vaccine high dose
RHC	recombinant hemagglutinin vaccine
IDTIV	intradermal trivalent influenza vaccine
HD	High dose
SD	standard dose
